# Coulomb-like elastic interaction induced by symmetry breaking in nematic liquid crystal colloids

**DOI:** 10.1038/s41598-017-16200-z

**Published:** 2017-11-21

**Authors:** Beom-Kyu Lee, Sung-Jo Kim, Jong-Hyun Kim, Bohdan Lev

**Affiliations:** 1 Department of Physics, Chungnam National University, 99 Daehak-ro, Yuseong-gu, Daejeon, 34134 Korea; 2 Bogolyubov Institute for Theoretical Physics of the NAS of Ukraine, Metrolohichna Str.14-b, Kiev, 03680 Ukraine; 3 Present Address: IBS Center for Soft and Living Matter, UNIST-gil 50, Ulsan, 689-798 Korea

## Abstract

It is generally thought that colloidal particles in a nematic liquid crystal do not generate the first multipole term called deformation elastic charge as it violates the mechanical equilibrium. Here, we demonstrate theoretically and experimentally that this is not the case, and deformation elastic charges, as well as dipoles and quadrupoles, can be induced through anisotropic boundary conditions. We report the first direct observation of Coulomb-like elastic interactions between colloidal particles in a nematic liquid crystal. The behaviour of two spherical colloidal particles with asymmetric anchoring conditions induced by asymmetric alignment is investigated experimentally; the interaction of two particles located at the boundary of twist and parallel aligned regions is observed. We demonstrate that such particles produce deformation elastic charges and interact by Coulomb-like interactions.

## Introduction

Liquid crystals (LCs) are anisotropic soft materials with continuous ground-state symmetry, susceptible to breaking under the influence of external factors. It is also possible to break the symmetry of LCs by introducing particles of specific materials into the host LC. The immersed particles break the symmetry of the LC alignments; such distortions can influence the LC alignment up to a distance of several times the particle size. Particles may be accompanied by topological defects such as boojum, Saturn-ring, and hyperbolic-hedgehog defects, depending on the surface conditions^1–3^. The combined structures of a particle and defect are usually in dipole-like or quadrupole-like configurations.

Furthermore, LC distortions induced by a combined particle–defect structure result in a new class of long-range interactions that do not occur in regular colloids. The behaviour of these interactions is similar to that of the electric dipole–dipole or quadrupole–quadrupole interactions in electrostatics^4,5^. In addition, these long-range anisotropic interactions result in the formation of structures such as linear and inclined chains^6–9^. A particle–defect structure in a dipole-like configuration also interacts with director deformations such as a boundary between twist and uniform alignment regions^10,11^. The magnitude and direction of the associated force are related to the orientation of the dipole-like configuration and the divergence of the director deformation. Both the magnitude and direction of the force vary when the dipole-like configuration moves in the deformed director field. Particles at the nematic liquid crystal (NLC)–air interface^12–14^ and in quasi-two-dimensional systems of thin NLC cells form 2D crystals^15–18^, while 3D crystal structures can also be constructed in the bulk^19^. Moreover, variations in the shape and structure of colloidal particles bring diverse phenomena^20,21^ and new methods of handling defects may introduce new possibilities for creating functional devices^22–24^.

Both the particle shape and the surface anchoring provoke symmetry breaking by a particle immersed in an LC^25,26^. When the anchoring is weak, the particle shape is the main factor causing the symmetry breaking. In contrast, both factors are significant in the case of strong anchoring, because there is an accompanying topological defect that influences the director distribution near the particle.

In the articles^23,24^ authors experimentally found for the first time the monopole Coulomb-like interaction between separate point topological defects – radial and hyperbolic hedgehogs in the vicinity of the fiber in NLC. In those papers there were no colloidal particles engaged in the process of interaction. In this report the monopole Coulomb-like interaction between separate colloidal particles is demonstrated in the specific geometry. In both cases, spatial director deformation mediates the interaction. In his book, de Gennes^27^ has shown that the deformation charge appears only with an existing external torque moment, and this torque can be viewed as the deformation charge^28–30^. It has a continuous value that depends on the anchoring energy and shape of the particles. As shown in the literature^24,25,30,31^, the appearance of the deformation charge is connected to the broken symmetry in the distribution of the director field around the particles.

The coat concept has been introduced to provide general description of many systems without considering their finer details^28,29^. A coat covering an isolated system consists of a particle and accompanying topological defect, and exhibits the same symmetry properties as the resulting director field around the particle and defect. The director distribution outside the coat does not contain any topological defects and contains only smooth variations. The shell of the coat is not an intrinsic characteristic of the particle, but depends on the field of the director that surrounds it. The fundamental interactions between the particles are determined by the symmetry of the director field on the coat. Overall, particle–particle interactions consist of all contributions of the overlapping director-field deformations that are caused by the surface anchoring of particles and substrate boundaries^29,30^.

In this study, we observed the motion of pairs of dipole-configured interacting particles at the boundary of two different alignment regions. We investigated their interacting behaviour by measuring the changing rate of the particle separation. Coulomb-like interactions appear to dominate when the particles are separated by more than several times the particle radius. Conversely, dipole-dipole-like interactions appear to play a major role when the particles are closer than several times the particle radius.

## Results

**Figure 1:**
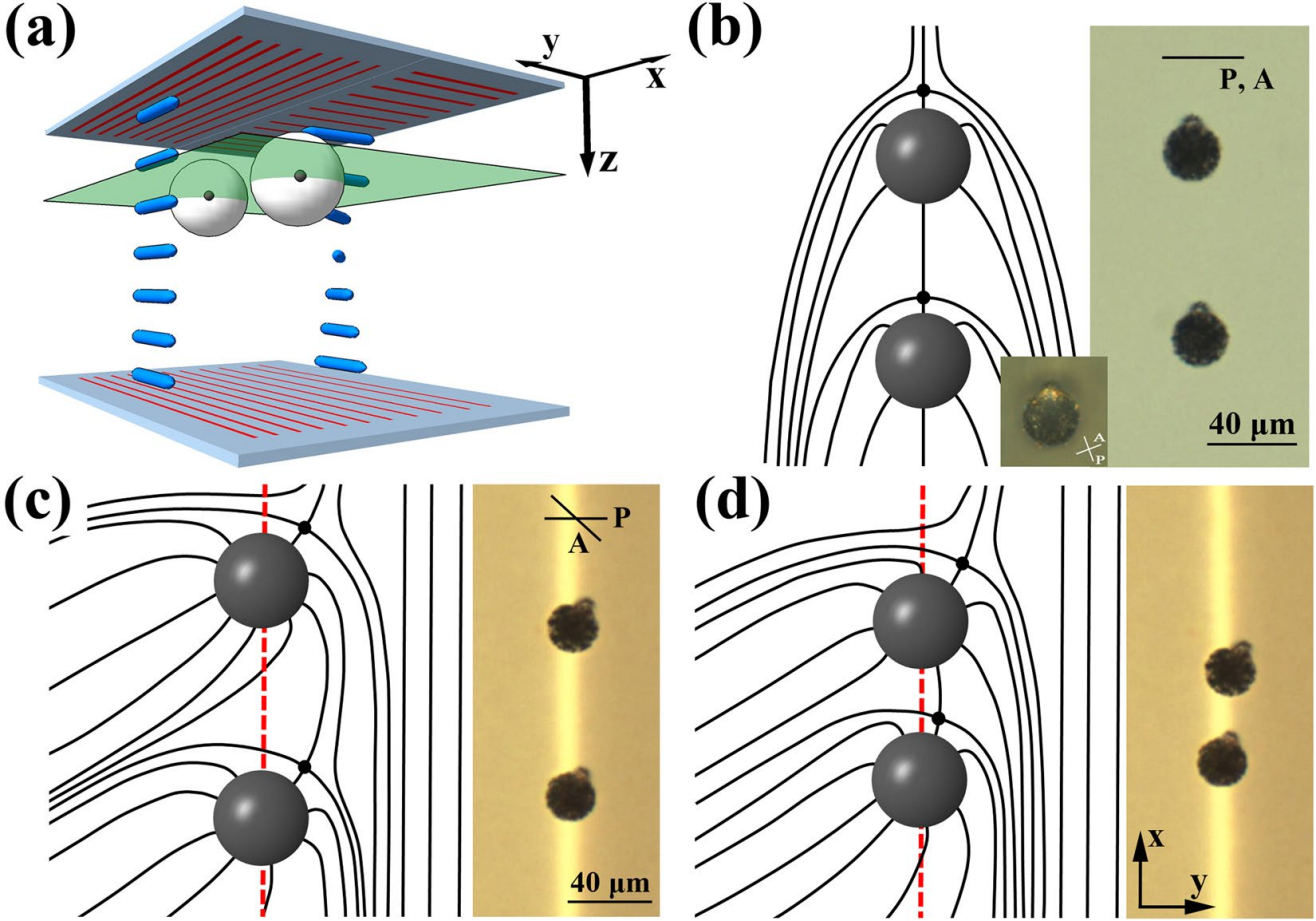
(**a**) Schematic of the cell structure and director configuration. The LC texture shows parallel- and twist-aligned regions. The particles are located at the boundary of the two regions. Each particle is accompanied by a hedgehog defect. The large white circles represent the particles, and each dark point in the circle is a point defect. The red lines indicate the alignment orientation on the substrates, and the thick blue lines indicate the directors. The green sheet is the viewing height of (**c**) and (**d**). (**b**) Two dipolar-configured particles that are in the uniformly aligned director field and a schematic diagram of the director field in the plane cut along the equator of the particles. Inset is the image of a dipolar-configured particle under crossed polarizers. Brightness of inset was adjusted for clarity. (**c**) Two dipolar-configured particles and a schematic diagram of the director field. The red dotted line is the boundary between the two regions. (**d**) The same particles as (**c**) at a closer distance. P and A in the figure correspond to the polarizer and analyser of the optical microscope, and the direction of the line indicates the polarization.

We used LC cells to observe the particles interactions experimentally. A substrate of a cell was patterned to create two regions of parallel and twist alignment, as shown in Fig. 1(a). The cell was injected with a mixture of NLC and micro-particles. Figure 1(b) shows two dipolar-configured particles in a director field uniformly aligned along the vertical direction. Figure 1(c),(d) show two dipolar-configured particles at the boundary of the deformed director field of (a). The director field configuration breaks the mirror symmetry along the line connecting the two particles in Fig. 1(b). However, there is a rotational symmetry with respect to the axis connecting the two particles, as well as mirror symmetry with respect to the planes passing through the line connecting the two particles. In contrast, in Fig. 1(c),(d), all the rotational and mirror symmetries, including the broken mirror symmetry along the line connecting the particles, are also broken in that director field configuration. We used a polarizing optical microscope to observe the change in distance between the two particles (Fig. 1(c),(d)). The texture of the boundary between the two neighbouring regions does not show defect line, but connected smoothly with changing director orientation at the patterned surface.

The direction of the dipole configuration is defined as the direction from the point defect to the particle centre. The orientations of the two dipole configurations are nearly parallel but not linear, as illustrated in Fig. 1(c),(d); this is different from the configuration in Fig. 1(b). Analysing the change in the particle separation helps understanding the interaction that drives the motion of the particles. Unless otherwise stated, all measurements were performed at room temperature.

**Figure 2:**
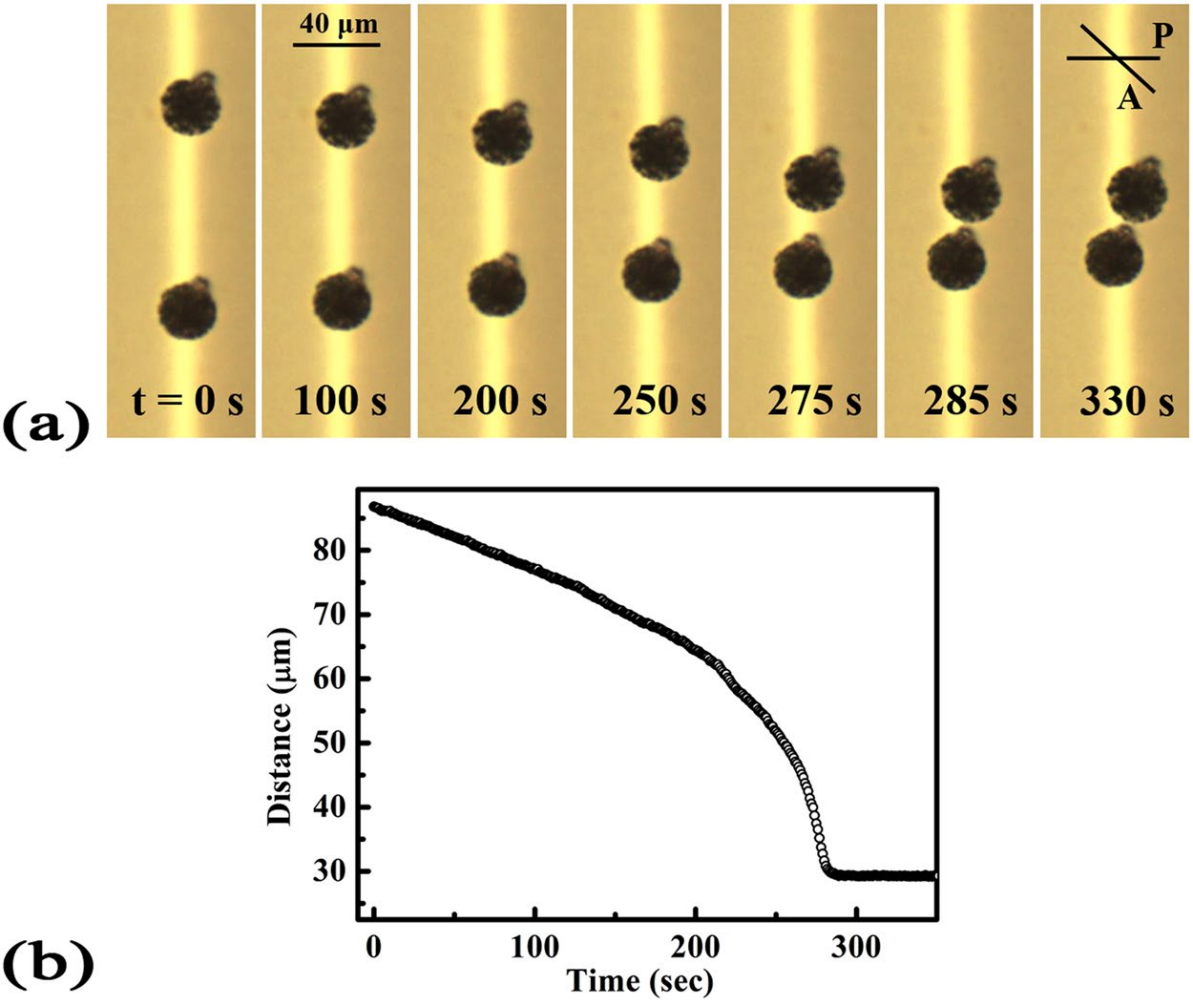
(**a**) Images of approaching particles at the boundary of two different alignment regions over time. The average particle radius is 12.3 µm. (**b**) The change in the particle separation as a function of time. Zero time corresponds to the start of the measurement. P and A in the figure correspond to the polarizer and analyser of the optical microscope, and the direction of the line indicates the polarization.

Two particles in the similar dipole orientation interact through the elastic deformation in the director field, so that the particle separation decreases with time, as in Fig. 2(a). Initially, the approaching speed is small, and it increases monotonically as the particles get closer, as in Fig. 2(b). At very small particle separations, their motion stops. The orientations of the dipoles also change slightly during the approach.

**Figure 3:**
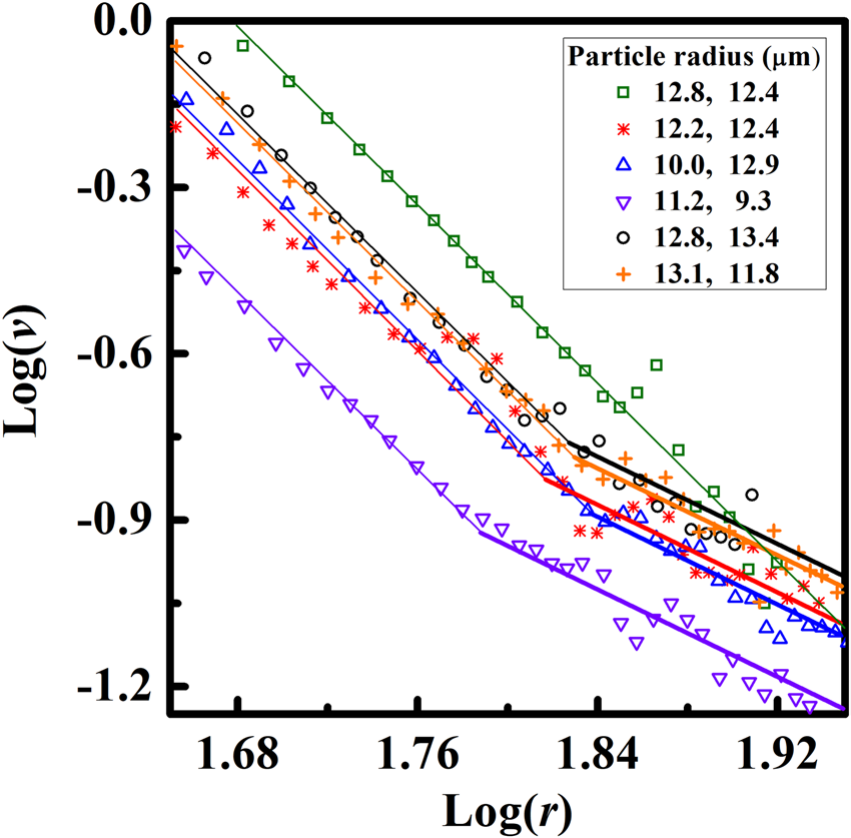
Experimental results obtained from observing the motion of several pairs of particles. The graph indicates the relationship between Log(*r*) and Log(*v*), where *r* is the particle separation and *v* is the approaching speed. The discrete symbols indicate the experimental results. The thick lines on the symbols indicate the matching with a slope of –2, and the thin lines indicate the matching with a slope −4. The top data set (empty green squares) is matched by a single line with a slope of −4, which indicates a dipole-dipole-like interaction. The other data are matched by two lines with slopes of −2 and −4. The fitted slopes to the experimental data are −3.89 ± 0.18 for genuine dipole-dipole-like interaction, –4.11 ± 0.25 in the range corresponding to dipole-dipole-like interaction at the boundary of the two aligned regions and −2.23 ± 0.14 in the range corresponding to Coulomb-like interaction respectively. The values after the symbols in the inset box show the radii of the two interacting particles. The units for distance (*r*) and speed (*v*) are µm and µm/sec, respectively.

Figure 3 shows the results of measuring the distance and approach speed between the two particles. The force between two Coulomb-like interacting particles is expressed as $a/{r}^{2}=bv$ ^32^, where *r* is the particle distance and *v* is the speed of the particle. The right-hand side corresponds to Stokes drag force, with b expressed as 6πγR, where γ is the viscosity and R is the particle radius. For Coulomb-like interactions, Log(*v*) is then linearly proportional to Log(*r*) with a slope of –2. Similarly, for the dipole-dipole-like interacting particles, Log(*v*) is linearly proportional to Log(*r*) with a slope of –4. The top green square symbol and line indicate the data obtained for the uniform planar alignment, which exhibit a genuine dipole-dipole-like interaction with a slope of –4 over the entire range. The other data set was obtained at the boundary of the two differently aligned regions, as in Fig. 2(a), and exhibits two distinct regions with slopes of –2 and –4. These pairs of particles interact mainly by Coulomb-like interactions at large separations and by dipole-dipole-like interactions at small separations. The crossover between Coulomb-like and dipole-dipole-like interactions occurs at a similar distance in all of these graphs. The crossover distance represents the point at which the Coulomb-like and dipole-dipole-like interactions are of equal strength. We use the crossover point to determine the value of the deformation charge. To confirm our approach, we also fit the presented data with linear functions. The genuine dipole-dipole-like interaction shows the slope of –3.89 ± 0.18 for various fitting ranges. In the range corresponding to dipole-dipole-like interaction at the boundary of the two aligned regions the slope is –4.11 ± 0.25, while in the range corresponding to Coulomb-like interaction the slope is –2.23 ± 0.14. The measured slope for Coulomb-like interaction seems to deviate from the expected value of –2. However, this is likely due to the dependence of the fitted slope on the fitting range and the large fluctuations in the measured data for the large distances where the interaction is weak.

## Discussion

We explain the origin of the deformation charge appearing under the experimental conditions as follows^3,28,29,33^. Let us consider a director-field deviation *δ* ***n*** from its ground state ***n***_0_. The texture in the absence of immersed particles corresponds to ***n***_0_. The director field ***n*** *(* ***r*** *)* is then described by ***n*** *(* ***r*** *)* = ***n***_0_ + *δ* ***n***, and satisfies |***δn*** *(* ***r*** *)*| ≪ 1 and ***n***_***o***_ ***∙δn(r)*** = 0. *δ* ***n*** satisfies the Euler–Lagrange equation *Δδ* ***n*** = 0 for infinitesimal *δ* ***n*** at a position far away from the particle. A particle immersed in an LC produces a director-field deformation that breaks the ground state symmetry^30^. The deviation from the ground state is difficult to determine near the particle for a non-linear elastic deformation response, although it can be determined at distances far from the particle using the aforementioned coat approach. At distances far from the particle, the deformation can be determined by taking into account the symmetry breaking of the director field at short distances^28,29,31^. Using the Euler–Lagrange equation, it is possible to expand *δ* ***n*** *(r)* in a multipole expansion^3,28,29^.

1 $$\delta {n}_{\mu }=\frac{{q}_{\mu }}{r}+\frac{{p}_{\mu }^{\alpha }{r}_{\alpha }}{{r}^{3}}+\frac{{Q}_{\mu }^{\alpha \beta }{r}_{\alpha }{r}_{\beta }}{{r}^{5}}+\ldots ,$$

where *µ* denotes the components in the direction perpendicular to the ground state; indices *µ, α*, and *β* run through all coordinate directions, and summation over repeated indices is assumed; ${q}_{\mu }$, ${p}_{\mu }^{\alpha }$, and ${Q}_{\mu }^{\alpha \beta }$ are the elastic monopole (charges), dipole, and quadrupole moments, respectively. The multipole expansion in Eq. (1) indicates that the director-field deviation induces a long-range effect. The validity range of each term may depend on several factors such as particle size, anchoring, and alignment. The deformations induced by two remote particles may overlap with each other. Deformation overlapping means that each particle feels the presence of the other particle, or, in other words, two particles with overlapping deformations interact by an elastic long-range interaction. In the case of strong anchoring, the non-linear solution for δ***n*** *(r)* displays an asymptotic behaviour near the particle. However, when the anchoring is weak, δ***n*** *(r)* is small, and the expansion in Eq. (1) is valid over the entire space^29^.

The torque in LC is related to the first term in Eq. (1), and applying an external torque ***Γ***_*ext*_ on the LC colloid is thought to be the only method to produce a deformation that is inversely proportional to *r* ^27^. In this work, we demonstrate that elastic monopoles can be induced by the influence of boundary conditions on the surface of the substrates and the particles. It is well known that NLCs transmit torques. A torque ***Γ*** acting on an NLC can be described by ${\boldsymbol{\Gamma }}=[{\boldsymbol{n}}\times \delta F/\delta n]$, where *F* is the free energy^27^. The deformation free energy $({F}_{def})$ is related to the director deformation and can be described using one-constant approximation as:^27^

2 $${F}_{def}=\frac{K}{2}\int dV[{({\boldsymbol{\nabla }}\cdot {\boldsymbol{n}})}^{2}+{({\boldsymbol{\nabla }}\times {\boldsymbol{n}})}^{2}],$$

where *K* is the elastic constant and *V* is the total volume.

The relationship between the torque from the deformation (***Γ***_*def*_) and the monopoles can be written as ${{\boldsymbol{\Gamma }}}_{def}={\boldsymbol{n}}\times \delta {F}_{def}/\delta n=4\pi K{\boldsymbol{q}}$ ^27^. The deformation decreasing in proportion to 1/*r* is related to the torque. ***Γ***_*def*_ is the torque inducing the elastic monopole ***q*** in an NLC. The particle will feel a deformation torque (−***Γ***_*def*_) with the elastic monopoles and particle rotation. Thus, at equilibrium, ***Γ***_*ext*_ = *−* ***Γ***_*def*_ is satisfied.

The external torque is estimated by taking into account the boundary conditions on the particle surface. The anchoring energy is related to the director orientation on the particle surface. Anchoring energy $({F}_{surface})$ may be expressed in Rapini–Papoular form:

3 $${F}_{surface}=\oint dSW(s){[{{\boldsymbol{n}}}_{{\boldsymbol{e}}}(s)\cdot {\boldsymbol{n}}(s)]}^{2},$$

where *W(s)* is the anchoring strength and ${{\boldsymbol{n}}}_{{\boldsymbol{e}}}(s)$ is the orientation of the easy axis on the particle surface *S*. The surface energy produces the torque ***Γ***_*surface*_.

4 $${{\boldsymbol{\Gamma }}}_{surface}=[{\boldsymbol{n}}\times \frac{{\boldsymbol{\delta }}{{\boldsymbol{F}}}_{{\boldsymbol{surface}}}}{{\boldsymbol{\delta }}n}]\approx 2\oint dSW(s)({{\boldsymbol{n}}}_{{\boldsymbol{e}}}\cdot {{\boldsymbol{n}}}_{{\boldsymbol{o}}})[{{\boldsymbol{n}}}_{{\boldsymbol{o}}}\times {{\boldsymbol{n}}}_{{\boldsymbol{e}}}]$$

A particle with a broken symmetry with respect to the plane perpendicular to ***n***_o_ and a broken symmetry with respect to at least one vertical symmetry plane yields non-zero integrals in Eq. (4). The torque is obtained from the condition ***Γ***_*surface*_ + ***Γ***_*def*_ = *0*. This situation is realised in our experiment. There, we have particles with different boundary conditions at the top and bottom surfaces. The director-field distribution on the left- and right-hand sides of the particle is also different in the vertical plane. There is a twist distribution in one half-region and a planar distribution in the other half-region. We can directly calculate the value of the deformation charge and compare it to that obtained from the experiment. In the deformed area, the particle surface experiences a torque that is produced by the distortion, the boundary conditions, and the dipole configuration.

The variable $\theta$ is introduced as the angle between the separation vector of the particles and the dipole, or the long axis of the deformation coat. The torque **Γ** is proportional to $sin\theta cos\theta$ ^27^, and the interaction energy (*U*_*int*_) can be expressed in a general form for Coulomb-like (*U*_*mm*_) and dipole-dipole-like (*U*_*dd*_) interactions^3^.

5 $${U}_{int}={U}_{mm}+{U}_{dd}=4\pi K\{\frac{-qq^{\prime} }{r}si{n}^{2}\theta co{s}^{2}\theta +\frac{pp^{\prime} }{{r}^{3}}(1-3co{s}^{2}\theta )\},$$

where *q* and *q*′ are monopole charges and *p* and *p*′ are dipole moments. Extremum values of this potential energy at a given *r* and variable $\theta$ satisfy the following condition:

6 $$co{s}^{2}\theta =\frac{1}{2}+\frac{3{{\rm{p}}}^{2}}{2{{\rm{q}}}^{2}{r}^{2}}\,\,{\rm{for}}\,q=q^{\prime} \,{\rm{and}}\,p=p^{\prime} $$

If the particle separation changes, the dipole moment orientation changes as well, while there is no variation when the distance remains constant. If the determined relation between the angle and distance is substituted into Eq. (5), the real interaction energy at all distances, which represents the monopole, or the deformation charge, can be obtained as ${U}_{int}={U}_{mm}+{U}_{dd}=-4\,\pi \,K({q}^{2}/4r+{p}^{2}/2{r}^{3})$. The crossover distance between the dipole-dipole-like interaction ${F}_{d}=-\partial {U}_{dd}/\partial r$ and the Coulomblike interaction ${F}_{m}=-\partial {U}_{mm}/\partial r$ is determined from the equality of the two forces. But we understand that this is an approximation and more precise approach should take into account the rotation of the radius vector between particles.

We obtain the crossover distance as ${r}_{c}=\sqrt{6}(p/q)=\sqrt{6}(\alpha /\rho )R$, where *p* = *αR* ^2^ and *q* = *ρR*. *R* is the radius. *α* is the coefficient of the elastic dipole moment with the value of 2.04 obtained using different ansatzes for the distribution of director field around the isolated particle^3^. *ρ* is the coefficient of the elastic monopole charge. We obtain *r*_*c*_ = 66.5 ± 2.6 µm from the data shown in Fig. 3. *q* becomes 11.4 µm with radius *R* = 12.3 µm and above *r*_*c*_ value. We then obtain *ρ*/*α* = 0.45 and estimate the coefficient *ρ* = 0.92. We can roughly estimate the value of *q* from the torque balance condition. The deformation torque is $4\pi Kq$ in the above, and the surface torque may be considered to be $2W\,4\pi {R}^{2}$ in Eq. (4). *q* is expressed as 2*WR* ^*2*^ */8* *K* for the assumption that one eight of the surface is not compensated in director field distribution and is effective to the torque for the anisotropy in director orientation. The calculated *q* value satisfies the above experimentally obtained *q* for K = 7 × 10^−12^ N and W = 2 × 10^−6^ J/m^2^ ^34,35^. This estimate indicates that even small portion of surface anisotropy can satisfy the experimental value of monopole charge.

Furthermore, we numerically calculate the monopole charge. The director orientation at the particle surface was calculated by dividing the surface into several dozens of points, and ***Γ***_*surface*_ was obtained from Eq. (4) by adding up all the contributions. The value of *q* can then be obtained from the torque balance condition. The calculation was simplified by several assumptions. The particle is assumed to be located at the exact boundary at a certain height. The director orientation on the particle surface is determined by linear interpolation between the nearest substrate of infinite anchoring and the particle surface of finite anchoring. The effect of point defect can be neglected for small size. The above values of the particle surface anchoring, particle size, and LC elastic constant were used. The calculated monopole charge is on the order of 1 µm. All values calculated using the described above simplifications and approximations are in qualitative agreement.

**Figure 4:**
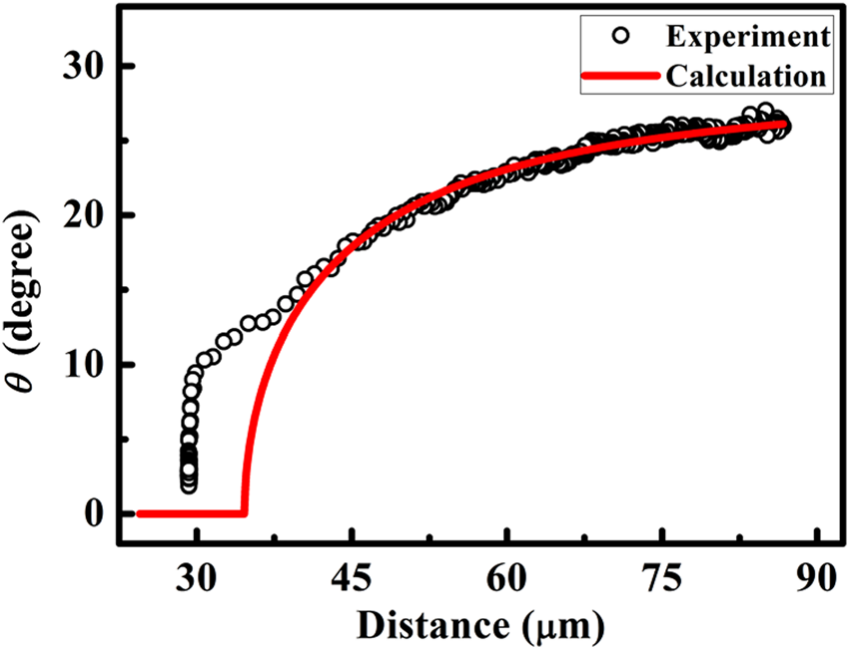
Change in *θ* as a function of the particle separation. *θ* is defined as the angle between the separation vector of the particles and the dipole. Plotted are the average values for the two particles. The symbols represent the experimental data from Fig. 2(a), and the line represents the data calculated from Eq. (6). To calculate *θ*, we used *ρ*/*α* = 0.9 and *R* = 12.3 µm. Moreover, we modified Eq. (6) to satisfy *θ* = 29° at large particle separations.

The orientation of the topological dipoles and the separation vector in Fig. 2(a) exhibits continuous rotation as the two particles approach each other. For large distances, the rotation of the topological dipole is significant. The rotating trend can be explained by Eq. (6). The change in *θ* is caused by the competition between the Coulomb-like and elastic dipole-dipole-like interactions that occur as the particle separation changes in uniform texture. At large particle separations, *θ* = 45° must be satisfied, while *θ* = 0° must be satisfied at particle separations less than the critical distance. Figure 4 presents the experimental and calculated change in *θ* as a function of the particle separation. If we use the values of the parameters in Eq. (6) as they are, the calculated and experimental curves deviate from each other. The experimental data indicates that *θ* = 29° at large particle separations. At particle separations >40 μm, the change in *θ* is described by *ρ*/*α* = 0.9. The fact that the experimental data at large particle separations is fitted for *θ* = 29°, and not *θ* = 45°, is due to the difference in the alignment conditions in the surrounding of the particles. *θ* = 45° corresponds to the ideal orientation of two particles interacting by Coulomb-like and dipole-dipole-like interactions in a uniform alignment. In the experiment, the particles are located in the middle of different alignment patterns, so that the orientation of the interacting particles is expected to deviate from the ideal situation. These issues seem to result in *θ* = 29° at large particle separations. The decreasing *θ* at small particle separations is due to the strong elastic dipole-dipole-like interaction compared to the Coulomb-like interaction. The discrepancies between the experimental and calculated results at small particle separations originate from the limitations of the theoretical approach. However, these results appear to be sufficient to indicate the existence of elastic Coulomb-like interactions.

In conclusion, we found a concrete example in which the surface of a particle induces a non-zero deformation charge in NLCs. In the experiment, we utilized varying alignment conditions on a substrate, which caused varying surface director orientation with broken symmetry on different parts of the particle. We expect that symmetric particles with varying director-field distributions in the bulk will produce monopoles as a result of the asymmetrical boundary conditions on the substrate surface and the particle. These boundary conditions on the surface of the particle and cell fully satisfy the necessary conditions for the existence of the deformation charge, with the electrostatic analogy described in the literature^21,24^. These results manifest the first proof of the presence of Coulomb-like interactions on the far distance in elastic media between separate colloidal particles

## Methods

### Liquid crystal cell

The LC cell consisted of two substrates that were prepared differently: one had an orientation alignment pattern and the other had a uniform alignment. The LC on the alignment pattern aligns into two perpendicular orientations in the substrate plane. A photo-alignment material, composed of a polyamic acid including azo-units in the main chain, was used as the alignment layer^36^. The azo-units respond to light by *cis*-*trans* isomerization. The layer aligns the director perpendicular to the linear polarization of the incident light in the substrate plane. The alignment material was irradiated with a 405 nm diode laser. The irradiated position was controlled by moving the substrate with translation stages. The polarization of the incident light was controlled by a linearly polarized light and a twisted nematic cell. The polarization was selected with the electric field in the LC cell matching the expected irradiated position. The other substrate was coated with a planar alignment material (AL-3046) and rubbed uniformly. The orientation of the two substrates was controlled to bring two regions with parallel and twist alignments. The ratio of the width (y-axis) of the twist region to that of the parallel region on the top substrate was 120:80 μm or 100:80 μm. The cell gap was 70 μm.

### Liquid crystal and micro-particles

The LC was 4-cyano-4′-pentylbiphenyl (5CB, from Merck), which exists in the nematic phase at room temperature. The density of 5CB is 1.01 g/cm^3^. The LC was mixed with a small amount of micro-particles. The micro-particles were made from polyethylene (GRYPMS, from Cospheric) and had a 10 ± 3 μm radius and a 1.0 g/cm^3^ density.

The micro-particles induced homeotropic anchoring and were accompanied by hedgehog or Saturn-ring defects in nematic phase^35^. We focused on particles in dipole configurations accompanied by hedgehog defects. The particles did not stick to either substrate due to both the small difference in density between the LC and the particles and the repulsive force between the particles and substrates^11^. The length of the pattern along the x-axis was sufficiently large, so that the alignment was assumed to be uniform.

### Image analysis

The difference in the motion of different particle pairs appears to stem from slight variations in the particle size and other factors, such as anchoring and surface conditions. The original data obtained from these experiments is evenly distributed over time. The particle separation changes very little during the initial stages of the experiments and, consequently, a large error is introduced in the particle-speed calculations. To overcome this issue, we slightly smoothed the raw data without obvious variation in data positions and interpolated the data to the evenly distributed particle separation data. The particle speed (y-axis data) was obtained by differentiating the particle separation (x-axis data) with respect to time in Fig. 3. In order to match the data with the lines of slope −2 and −4, we prepared the lines of slope −2 and −4 and adjusted the intercept of the Log(*v*) axis.
